# Exosomes: A Friend or Foe for Osteoporotic Fracture?

**DOI:** 10.3389/fendo.2021.679914

**Published:** 2021-06-21

**Authors:** Zhimin Yang, Wenchao Zhang, Xiaolei Ren, Chao Tu, Zhihong Li

**Affiliations:** ^1^ Department of Orthopaedics, The Second Xiangya Hospital of Central South University, Changsha, China; ^2^ Hunan Key Laboratory of Tumor Models and Individualized Medicine, The Second Xiangya Hospital of Central South University, Changsha, China

**Keywords:** exosomes, osteoporosis, fracture healing, bone physiology, osteoporotic fracture

## Abstract

The clinical need for effective osteoporotic fracture therapy and prevention remains urgent. The occurrence and healing of osteoporotic fracture are closely associated with the continuous processes of bone modeling, remodeling, and regeneration. Accumulating evidence has indicated a prominent role of exosomes in mediating multiple pathophysiological processes, which are essential for information and materials exchange and exerting pleiotropic effects on neighboring or distant bone-related cells. Therefore, the exosomes are considered as important candidates both in the occurrence and healing of osteoporotic fracture by accelerating or suppressing related processes. In this review, we collectively focused on recent findings on the diagnostic and therapeutic applications of exosomes in osteoporotic fracture by regulating osteoblastogenesis, osteoclastogenesis, and angiogenesis, providing us with novel therapeutic strategies for osteoporotic fracture in clinical practice.

## Introduction

Bone is an essential component of the musculoskeletal system, providing structural support for tendons and ligaments as well as protection to vital internal organs. Bone fracture is a worldwide public health problem and often leads to dire consequences, exerting a strong threat on life quality and even mortality (1). It results from injury, or a mild stress under certain pathological conditions that weakens the bones, like osteoporosis (OP), bone cancer, or osteogenesis imperfecta (2). In particular, OP is a major systemic musculoskeletal disorder characterized by deterioration of micro-architecture and bone loss, with an inclination to high risks of bone fragility and even fracture (3). As a matter of fact, osteoporotic fracture accounts for a large proportion of all fractures. Osteoporotic fracture-related impaired healing, especially non-union, is another critical problem, resulting in prolonged treatment and aggravated socio-economic burden.

Exosomes were first identified in 1981 as exfoliated membranes by Trams (4). At present, exosomes are defined as cell-derived spherical lipid bilayer vesicles (EVs) with a diameter around 40-160nm (5). Exosomes can be secreted by nearly all sorts of cells and are found in various biological fluids, such as plasma, serum, and cerebral spinal fluid (6). Previous studies have demonstrated that the constituents of exosomes are macromolecules including DNAs, RNAs (mRNA, microRNA, and other non-coding RNA), proteins, lipids, and cytokines (7, 8). Size and content are two major manifestations of the heterogeneity for exosomes, which vary with cellular origin, metabolic status, and environment of cells (5). Size heterogeneity is beneficial to distinguish exosomes and other EV subtypes. This property is also highly relevant to exosome isolation. Nowadays, some researchers usually use differential centrifugation to purify exosomes, but these exosome pellet fractions contain more than just exosomes. Hence, we require modified novel techniques for exosome purification, which is directly associated with the results of many exosomes-related experiments. Content heterogeneity determines the functions of exosomes since exosomes can convey their contains into recipient cells *via* endocytosis, receptor- ligand interaction, or fusion of membranes (9), thus mediating lots of responses of these recipient cells. Moreover, numerous studies have demonstrated that exosomes were obviously related to immune responses (10), pregnancy (11), and the occurrence and development of many disease, such as cardiovascular disease (12), central nervous system-associated diseases (13), and cancer (9). For example, exosomes secreted by apoptotic vascular endothelial cells were rich in miR-126, which could suppress the apoptosis of endothelial cells by activating chemokine ligand 12 (CCL12). Moreover, miR-126 containing exosomes inhibited the penetration of macrophages into the blood vessel wall, thereby stabilizing the hardened plaque and exerting the anti- atherosclerosis effect (14). Exosomes derived from prostate cancer cells could induce neoplastic reprogramming and tumor formation of adipose stem cells *via* transferring their cargos, like miR-125b, miR-130, miR-155, HRas, and Kras mRNAs (15). Apoptotic glioblastoma cells could secret spliceosomal proteins such as RBM11 and small nuclear RNAs (snRNAs) containing exosomes to modify mRNA splicing (MDM4, CCND1) of recipient cells, resulting in tumor aggressiveness and drug resistance (16). Significantly, translation of more comprehensive understanding of exosomes in various diseases into diagnosis and therapeutic applications has already occurred. First, the intrinsic properties of exosomes have exhibited their great potential in diagnosis of multiple diseases. Lewis et al. presented a simple method which integrated and analyzed exosomes as well as other extracellular vesicles directly from whole blood, plasma, or serum onto an AC electrokinetic microarray chip. They detected the samples from pancreatic ductal adenocarcinoma (PDAC) patients as well as healthy objects, finding that glypican-1 and CD63 were useful biomarkers to predict the occurrence of PDAC. These researchers also developed a bivariate model to detect PDAC with 99% sensitivity and 82% specificity (17). In addition, exosomes have been implicated as therapeutic targets in many fields, with potential utility in delivering therapeutic payloads directly to the desired place (18). Exosomal microRNA (miRNA) could target mRNA and modify related gene expression in the recipient cells, and some laboratories have attempted to use exosomes for the delivery of miRNA or small interfering RNA (siRNA) to treat mammary carcinoma (19), glioma (20), and pancreatic cancer (21). Moreover, clinical-grade MSC-derived exosomes with Kras^G12D^ siRNA payload (iExosomes) have been applied to the treatment of pancreatic cancer in various animal models (22). Taken together, exosomes play an important role in mediating a variety of physiological and pathological processes through the special intracellular communication, which provides us a promising avenue to conquer numerous disorders, including osteoporotic fractures (23).

In this review, we aimed to illustrate the relationship between the exosomes and osteoporotic fracture, while referring to the complicated occurrence and healing processes. Moreover, we summarized the applications of exosomes in preventing and treating osteoporotic fracture, which may be an invaluable tool for the intervention of osteoporotic fracture and other related musculoskeletal disorders.

## Bone Physiology and Exosomes

Bone consists of minerals and organic material. The minerals include crystals of hydroxyapatite Ca_10_(PO_4_)_6_(OH)_2_ and other ions, while the organic material comprises osteogenic cells and extracellular matrixes like collagen fibers (24). Osteogenic cells mainly include three types of cells: the osteoblasts, osteoclasts, and osteocytes (25). In fact, exosomes exerted a dominant effect on bone turnover and remodeling by mediating these important elements of bone. On the one hand, exosomes played a key role in the process of production of bone extracellular matrixes. For instance, the exosomes membrane rich in phosphatidylserine (PS) participated in the formation of hydroxyapatite crystal during osteogenesis, a process that was also accelerated by the calcium stored in exosomal annexins and the phosphate generated by exosomal ATPases, nucleotidases, phosphatases, pyrophosphatases, and membrane transporters (24). On the other hand, exosomes acted as messengers to mediate signals transmitting in the same cluster (autocrine) or different cluster (paracrine) of osteogenic cells, thus participating in their differentiations and activations. Wei et al. found let-7 was contained in both osteoblast precursors and differentiated osteoblasts-derived exosomes, which could promote osteogenesis through mediating high-mobility group AT-hook 2 (HMGA2) and Axin 2 (26). Another study by Li et al. showed osteoclasts-derived exosomal miR-214-3p suppressed osteoblastic bone formation (27). Further, bone modeling and remodeling were tightly correlated with the endocrine system. And parathyroid hormone (PTH), estrogen hormone, and glucocorticoid were important factors in mediating the bone microenvironment for osteoanabolism (28). However, the specific relationships between exosomes and steroid or protein hormones in bone physiology is still not fully understood and needs further exploration. Overall, exosomes exert an indispensable role in these processes and future research analyzing their properties and functions may help to build a multi-targeted system to maintain the balance of bone resorption and formation.

## Relationships Between Exosomes and the Occurrence of Osteoporotic Fracture

Bone loss and a fall bias lead to great susceptibility to fractures for the aging and menopausal population (29). Clinically, osteoporotic fractures are common, have become a serious public health issue worldwide, and cause an ever-increasing burden to the healthcare system. OP has an obvious clinical and public health impact which is estimated to affect 75 million people worldwide (30). Moreover, the number of annual incident fragility fractures is about 9 million (31).

Mechanically, OP is a result of excessive bone resorption and inadequate formation of new bone upon aberrant activities of osteoclasts and osteoblasts (32). Under normal circumstances, bone metabolism follows the strict control of various factors. And among them, receptor activator of NF-κB (RANK)/receptor activator of NF-κB ligand (RANKL), Wnt/β-catenin, and Jagged1/Notch1 are the three best studied pathways which exert a strong influence on bone mass density (33). Strikingly, exosomes have been widely studied for their roles in OP, which make great contributions to the imbalance of osteoblasts and osteoclasts, hence promoting the prevalence of fragility fracture. Therefore, we comprehensively reviewed the role of exosomes in OP initiating bone fracture and presented their potential in retarding the occurrence and progress of osteoporotic fracture.

### The Functions of Exosomes in OP

**Figure 1:**
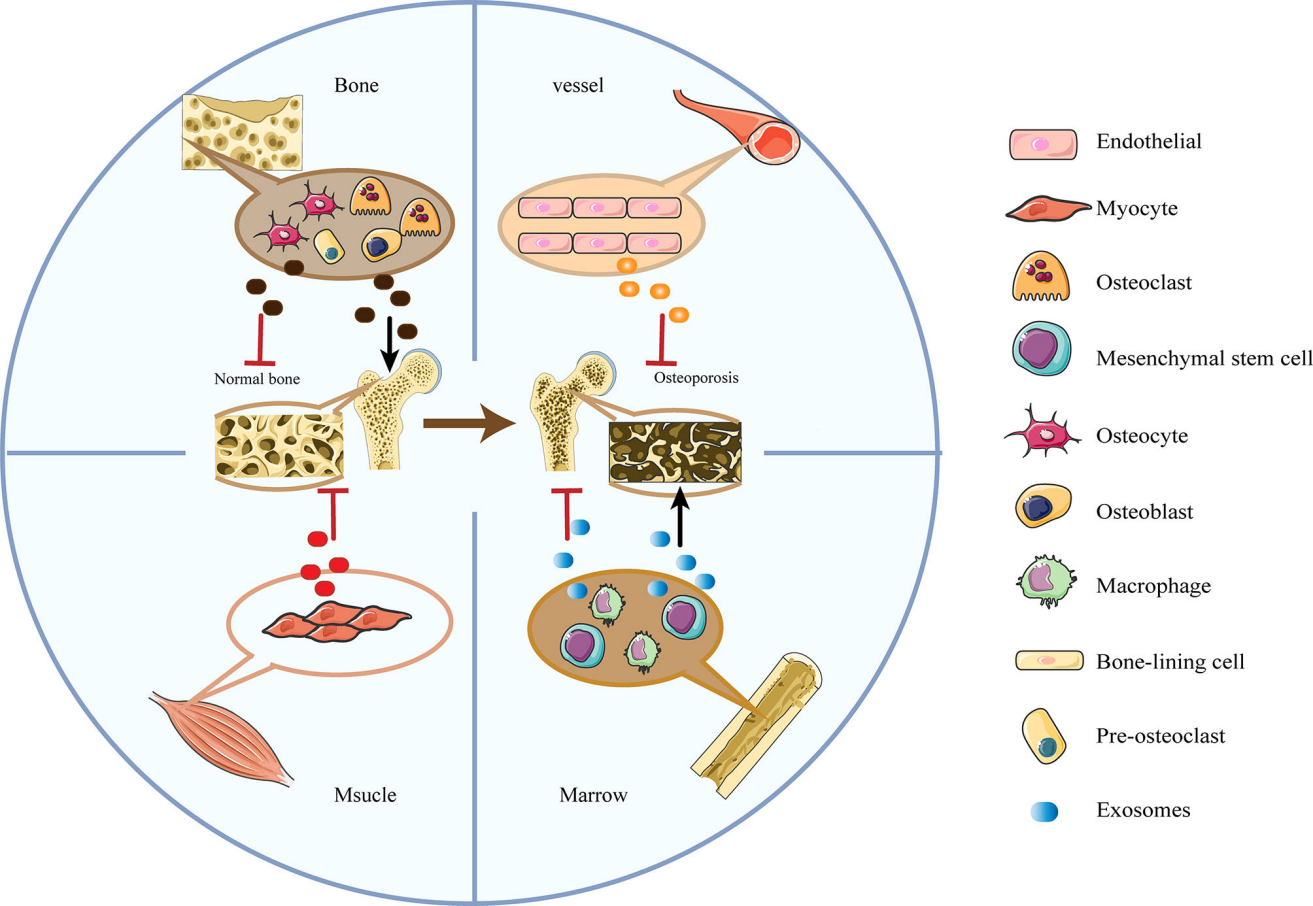
The diplex functions of bone-related cells-derived exosomes in OP. Exosomes secreted by osteoblasts, osteoblasts and mesenchymal stem cells have bilateral effects in promoting and suppressing OP; Exosomes derived from myocytes and vascular endothelial cells mainly inhibit the process of OP.

As mentioned above, exosomes functioned as “the carrier pigeons of the cell” in intercellular communication and materials exchange to mediate a series of responses of adjacent or distant recipient cells (34, 35). Multiple studies have shown that bone-related exosomes were tightly associated with bone modeling and remodeling (27, 36) by transferring biologically essential molecules to interfere with the activities of osteoblasts and osteoclasts (**Figure 1**). Besides, the circulatory exosomes possessed pathophysiological functions in the development of senile OP and thus would be helpful for its diagnosis and therapy.

#### Circulatory Exosomes in OP

A large number of reports detected the main differences of serum-derived exosomes (SDEs) between OP or osteopenia and normal bone mass by related techniques to get a more comprehensive understanding of the functions of exosomes in OP (**Table 1**). Xie et al. identified 1371 proteins from SDEs, especially, 585 differentially expressed proteins (DEPs) of OP. These DEPs not only participated in inhibiting integrin-related activation and function of osteoblasts, but also strengthened the capacity of osteoclasts. Notably, the integrin β_1_ (ITGβ_1_), integrin β_3_ (ITGβ_3_), and hematopoietic progenitor cell antigen CD34 (CD34) were three representatives of downregulated proteins, which acted as hubs in suppressing bone mineralization of osteoblasts (37). Chen et al. utilized the PLAGH Hip Fracture Database, finding a total of 45 significant DEPs and verifying four exosomal proteins, namely PSMB9, PCBP2, VSIR, and AARS, with an AUC of 0.805 in the classification of OP (38). Zhang et al. investigated the functions of transfer RNA‐derived fragments (tRFs), a novel type of small non‐coding RNAs derived from tRNAs contained in plasma exosomes in OP. They found 29 differentially expressed tRFs, which played an active role in some important pathways related to OP, including Wnt, PI3K‐Akt, MAPK, TGF‐β, and calcium signaling pathway. More importantly, plasma exosomal tRF‐25‐R9ODMJ6B26 (tRF‐25), tRF‐38‐QB1MK8YUBS68BFD2 (tRF‐38), and tRF‐18‐BS68BFD2 (tRF‐18) were at highly-expressed levels in OP patients compared to normal controls and were utilized to develop a more accurate model for OP diagnosis with an average AUC of 0.815 (39). Furthermore, a study by Teng et al. detected 393 differentially expressed lncRNAs and found co-located mRNAs were highly enriched in OP-associated processes, such as MAPK pathway, insulin secretion, cellular response to metal ions, fucosylation, and proteolysis (40). More importantly, Shao et al. isolated serum exosomes from menopausal females with or without OP and detected 191 aberrant miRNAs, which were related to Wnt, MAPK, and Hippo pathways (41). All of these findings offered forceful evidence to illustrate exosomes from the circulation could be potential biomarkers of evaluating bone status for OP diagnosis and analyzing the results of therapy.

**Table 1:** Exosomes from circulation in OP.

Origin of exosomes	Detected technique	Exosomes contains	Main difference	Reference
SDEs of patients with 31 osteoporosis, 46 osteopenia, and 62 normal volunteers.	TMT-based quantitative MS	Proteins	1,371 proteins were identified with an overlap of 1,160 Gene IDs among the ExoCarta proteins. 585 osteoporosis differentially expressed proteins were detected (255 upregulated and 360 downregulated).	(37)
Plasma exosome of patients with 30 osteoporosis, 10 osteopenia, and 20 normal controls	MS	Proteins	2351 proteins were identified in all groups, and 45 differentially expressed proteins were identified in the discovery dataset	(38)
Plasma exosome of patients with 40 osteoporosis, and 40 healthy controls	Small RNA sequence	tRFs	Found 288 total tRFs and 29 differentially expressed tRFs (11 upregulated and 18 downregulated)	(39)
SDEs of 9 elderly patients with fracture and 9 age-matched patients without fracture at the age between 60 and 90 years old	RNA-Seq experiments	LncRNAs	Detected 393 differentially expressed lncRNAs (296 upregulated and 97 downregulated)	(40)
SDEs of 6 menopausal females without osteoporosis and 12 menopausal females with osteoporosis	miRNA high-throughput sequencing	MiRNA	191 aberrant miRNAs were found in the group of menopausal females with osteoporosis (72 upregulated and 121 downregulated)	(41)

lncRNAs, long non-coding RNA; MS, mass spectroscopy; SDEs, serum-derived exosomes; TMT, tandem mass tag; tRFs, transfer RNA‐derived fragments.

#### Bone-Related Cell-Derived Exosomes in OP

Bone-related cells like osteoblasts, osteoclasts, and endothelial cells, could achieve mutual interaction through exosomes, the cell-cell communicators (**Table 2**). Over the past few years, many researchers have drawn attention to the relationship between bone-related cells-derived exosomes and OP. First, some laboratories have extensively investigated exosomes derived from bone-related cells by using techniques including Nanoparticle Tracking Analysis (NTA) (69), Transmission electron microscopy (TEM) (50), western blotting (WB), immuno-EM, or bead-based fluorescence-activated cell sorting (FACS) (63). More significantly, these scientists not only identified the classical bone-related cell-derived exosomal markers of OP, but also revealed the ability of exosomes to mediate osteogenic cells differentiation and activity as well as matrix formation in OP. Overall, this evidence indicated the essential role of bone-related cell-derived exosomes in OP and provided us serviceable tools in the treatment of OP.

**Table 2:** Bone-related cells derived exosomes in OP.

Origin of exosomes	Exosomes contains	Recipient cell	Involved pathway	Function	Reference
BM- MSCs	Not referred	Osteoblasts	Not referred	Promoted osteoblasts proliferation and inhibited cell apoptosis	(42)
BM- MSCs	Not referred	Osteoblasts	MAPK pathway	Promoted osteoblasts differentiation	(43)
BM- MSCs	MiR-196a	Osteoblasts	ALP, OCN, OPN and Runx2	Promoted osteoblasts differentiation, activation and proliferation	(44)
BM- MSCs	MiR-150-3p	Osteoblasts	Not referred	Promoted osteoblasts proliferation and differentiation	(45)
BM- MSCs	MiR‐181a	Osteoblasts	TGF- and Wnt signaling pathways	Promoted osteoblastic differentiation	(46)
BM- MSCs	MiR‐218	Osteoblasts	Wnt signaling pathways	Accelerated osteoblasts differentiation and mineralization	(47)
BM- MSCs	LncRNA MALAT1	Osteoblasts	MiR-34c/SATB2 axis	Alleviated osteoporosis	(48)
BM- MSCs	Let‐7	Osteoblasts	HMGA2	Increased osteogenesis and bone formation	(26)
BM- MSCs	MiR-29a	HUVECs, osteoblasts, osteoclasts	PCAF-mediated RANKL and CXCL12 or Frizzled 4	Promoted angiogenesis and osteogenesis and inhibited osteoclastogenesis.	(49)
BM- MSCs	Not referred	BM- MSCs	Not referred	Promoted the proliferation and osteogenic differentiation of BM-MSCs	(50)
hiPSC-MSCs	Not referred	BM- MSCs	Not referred	Enhanced angiogenesis and osteogenesis	(51)
BM- MSCs	MiR-186	BM- MSCs	Hippo signaling pathway	Promoted osteogenesis	(52)
BM- MSCs	Not referred	BM-MSCs, osteoblasts	Wnt/β-catenin signaling	Restored the function of BM-MSCs	(53)
BM- MSCs	MiR‐31a‐5p	Osteoblasts, osteoclasts	SATB2 and E2F2 pathways; RhoA pathway	Reduced osteoblastogenesis and promote osteoclastogenesis	(54)
BM- MSCs	MiR‐148a	Osteoclasts	V‐maf musculoaponeurotic fibrosarcoma oncogene homolog B	Promoted osteoclasts differentiation	(55)
BM- MSCs	MiR-21	BM- MSCs	Targeted SMAD7	Inhibited osteogenesis	(56)
Osteoblasts	MiR-677-3p	BM- MSCs	Increase AXIN1	Enhanced BM-MSCs differentiation	(57)
Osteoblasts	MiR-378	BM- MSCs	PI3K/Akt signaling pathway	Activated the glucose-mediated osteogenic differentiation	(58)
Osteoblasts	RANKL	Osteoclasts	RANKL-RANK	Led to osteoclasts information	(59)
Osteoblasts	MiR-30d-5p	Osteoblasts	RUNX2	Suppressed osteoblasts differentiation	(57)
Osteoblasts	MiR-133-3p	Osteoblasts	RUNX2	Suppressed osteoblasts differentiation	(57)
Osteoblasts	MiR-140-5p	Osteoblasts	BMP-2	Diminished osteoblast activity	(60)
Osteoblasts	Not referred	BM- MSCs	Not referred	Inhibit BM-MSCs differentiation	(61)
Osteoclast precursors	Not referred	Osteoclasts	Vitamin D-dependent pathway	Promote osteoclasts formation	(62)
Osteoclasts	RANK	Osteoclasts	RANKL-RANK	Inhibited osteoclastogenesis	(63)
Osteoclasts	MiR-214	Osteoblasts, osteoclasts	EphrinA2/EphA2, ATF4; PI3K/Akt pathway	Inhibited osteoblastogenesis, promoted osteoclastogenesis	(27, 64)
Osteocytes	MiR-218	Osteoblasts	Not referred	Promoted osteoblastic differentiation	(65)
Muscle	MiR-34a-5p	BM- MSCs	Sirt1	Induced BM-MSCs senescence	(66)
Endothelial cells	MiR-155	Osteoclasts	Spi1, Mitf, Socs1	Suppressed osteoclasts differentiation and activation	(67)
Endothelial cells	Not referred	Osteoblasts	ferroptosis	Rescued the glucocorticoid-induced osteogenic inhibition of osteoblasts	(68)

AXIN1, axis inhibition protein 1; BMP-2, bone morphogenetic protein 2; hiPSCs, human induced pluripotent stem cells hiPSC-MSC-Exos; HMGA2, high-mobility group AT-hook 2; HUVECs, human umbilical vein endothelial cells ; Mitf, microphthalmia-associated transcription factor; RUNX2, runt-related transcription factor 2; Socs1, suppressor of cytokine signaling 1.

##### MSCs-Derived Exosomes in OP

MSCs are pluripotent stem cells with the capability to proliferate extensively and maintain the potential to differentiate into various types of cells (70). In 2010, MSCs-derived exosome was first isolated from conditioned medium of human embryonic-derived MSCs (hESC-MSCs) (71). Nowadays, MSCs have been identified as the most ferocious producer of exosomes (72). Moreover, Chen et al. detected more than 850 gene products and 150 miRNAs in MSCs-derived exosomes (73, 74), indicating their potential clinical efficacy for a variety of diseases, including OP. In fact, a considerable amount of research has confirmed that BM-MSCs-derived exosomes could improve OP by triggering osteoblasts proliferation, differentiation, and activation as well as inhibiting cell apoptosis (42, 43) through miR-196a (44), miR-150-3p (45), miR‐181a (46), miR‐218 (47), lncRNA MALAT1 (48), and let‐7 (26). In addition, miR-29a loaded in BM-MSCs-derived exosomes could not only promote angiogenesis and osteogenesis but also restrain osteoclastogenesis, which was down-regulated in the aged populations, providing us a novel way to improve therapeutic strategy for OP (49). BM-MSCs-derived exosomes also have been identified as inducers to motivate undifferentiated MSCs toward osteogenic differentiation both *in vivo* and *in vitro* (50–52), showing its potential as a bone regenerative drug for OP. Moreover, Zuo et al. expounded that BM-MSCs-derived exosomes could promote bone formation by banishing reactive oxygen species (ROS), assisting in DNA repair, rescuing cell (mainly osteoblasts) proliferation and differentiation ability, suppressing the senescence-related protein and adipogenic gene expression, and accelerating osteogenic expression, thus ameliorating OP (53).

However, some studies also showed that BM-MSCs-derived exosomes could be helpful to OP with the function of promoting maturation and activity of osteoclasts. Previous studies have proven that miR‐31a‐5p was over-expressed in aged BM-MSCs- derived exosomes, which could reduce osteoblastogenesis by the SATB2 and E2F2 pathways and promote osteoclastogenesis by the RhoA pathway (54). Hence miR‐31a‐5p contained in BM-MSCs exosomes would lead to osteoporotic bone loss in aging bone tissue. Cheng et al. also detected that miR‐148a from BM-MSCs-derived exosomes could stimulate osteoclasts differentiation *via* targeting V‐maf musculoaponeurotic fibrosarcoma oncogene homolog B (55). Besides, a recent study by Jiang and colleagues demonstrated miR-21 was at a high level in BM-MSCs derived exosomes from OP patients, which inhibited osteogenesis by targeting SMAD7 (56).

Therefore, exosomes secreted by BM-MSCs are capable of mediating proliferation and differentiation of osteoblasts and osteoclasts, suggesting a novel method to improve therapeutic strategy for OP. For instance, researchers have successfully used exosomes from processed BM-MSCs, like miR-935-modified BM-MSCs and circ-Rtn4-modified BM-MSCs, to treat OP (75, 76).

##### Osteoblasts-Derived Exosomes in OP

Paracrine/autocrine in communication between osteoblasts and other bone-related cells in the field of OP has provided us with new knowledge and evoked further detection in this area. Ge et al. isolated osteoblasts-derived exosomes and analyzed their cargos, finding they were tightly combined with osteogenesis (57). Consistently, accumulating studies indicated that the contents of osteoblasts-derived exosomes, like miR-677-3p (59) or miR-378 (58), could accelerate osteogenesis, while RANKL (61), miR-30d-5p, miR-133-3p (59), and miR-140-5p (60) from osteoblast-derived exosomes possessed the ability to suppress this progress. Niedermair et al. recently revealed osteoblasts-derived exosomes from OP patients could also hamper osteogenic differentiation of BM-MSCs (77). Therefore, it remains a mystery for us as to what the specific functions of osteoblasts-derived exosomes in OP are.

##### Osteoclasts-Derived Exosomes in OP

Osteoclasts-derived exosomes have positive as well as negative feedback in the progress of osteogenesis, which played dual roles in OP. A recent study showed that exosomes secreted by osteoclast precursors facilitated vitamin D-dependent osteoclast formation in whole mouse marrow cultures, while exosomes from osteoclast-enriched cultures suppressed osteoclastogenesis in the same cultures which referred to an important factor-RANK (62). Furthermore, miR-214-containing exosomes from osteoclasts have been reported to have a significant effect on osteogenesis. On one hand, it could restrain osteoblasts *via* targeting EphrinA2/EphA2 (63) and activating transcription factor 4 (ATF4) (27). On the other hand, it could promote osteoclastogenesis through the PI3K/Akt pathway (64). Therefore, miR-214-3p may be a potential clinical target to reverse established OP. In fact, another study performed by Zhu et al. also found that the magnetic hydroxyapatite scaffold (MHA) facilitated osteoblasts’ proliferation in a model of OP through altering the osteoclasts-derived exosomal cargos and suppressing exosomes intussuscept by osteoblasts (78), indicating exosomes could act as tools to modify the interactions of bone cells or direct drugs to treat OP.

##### Osteocytes-Derived Exosomes in OP

Osteocytes are vital components of bone tissue that originated from osteoblasts and play multifaceted roles in bone remodeling. Emerging studies found osteocytes-derived exosomes could enter into circulation and carry some miRNAs, such as miR-29, miR-484, and miR-221 (79). Besides, Qin et al. offered evidence that exosomes from osteocytes could co-localize with the nucleus of MC3T3-E1 cells and reduce osteoblastogenesis. More significantly, they expounded the specific mechanism that myostatin possessed the ability to suppress osteocytes-derived exosomal miR-218, which was an unprecedented method for muscle-bone communication (65).

##### Muscle-Derived Exosomes in OP

Muscle has a mechanical crosstalk with bone. As aforementioned, muscle is identified as a secretory endocrine organ that takes part in biochemical interplay and influences the functions mutually. Bone-derived factors compromise fibroblast growth factor (FGF)-2, prostaglandin E2, osteocalcin, and sclerostin, while the muscle secreted factors are described as “myokines” (80), such as myostatin, interleukin (IL)-6, irisin, and RANKL. Recently, Fulzele et al. found that miR-34a-5p was overexpressed in muscle and muscle-secreted exosomes with aging and with myoblast exposure to oxidation (66). Further research showed that muscle-derived exosomes containing miR-34a could suppress Sirt1 expression in BM-MSCs and accelerate BM-MSCs senescence (81). The evidence represented a potential pathway by which muscle could affect bone physiology and provide us a different perspective to understand OP.

##### Endothelial Cells-Derived Exosomes in OP

Blood vessels occupy vital positions in bone homeostasis. Endothelial cells are stationed at the inner layer of vascular vessels, which actively participate in the progress of internalizing and secreting substances like exosomes. Song et al. found that miR-155 loaded in endothelial cells-derived exosomes suppressed osteoclasts’ differentiation and activation by several targets, like Spi1, microphthalmia-associated transcription factor (Mitf), and suppressor of cytokine signaling 1 (Socs1) (67). Furthermore, endothelial cells-derived exosomes could rescue the glucocorticoid-induced osteogenic suppression of osteoblasts by inhibiting ferritinophagy-dependent ferroptosis (68). Hence, endothelial cells-derived exosomes might be promising and biocompatible nanomedicine for OP.

##### Macrophages-Derived Exosomes in OP

Osteal macrophages are a subtype of bone-resident macrophages, which are close to the bone surface and adjacent to osteoblasts, with the function of mediating bone formation (82). Current knowledge about macrophage-derived exosomes is limited. A study by Wei et al. showed that BMP2/macrophage-derived exosomes could up-regulate the expression of osteogenesis-associated genes such as ALP, Runx2, BMP-2, BMP-7, and osteopontin, playing essential roles in OP (83). Collectively, the explicit mechanism of the macrophage-derived exosomes in OP remains unclear. However, with more in-depth study, it may show great potential acting as the therapeutic target for OP in the future.

## Relationships Between Exosomes and Osteoporotic Fracture Healing

Bone is a unique form of tissue that can heal without a fibrous scar (84). Similar to the process of bone remodeling, bone regeneration is highly orchestrated and precisely controlled, and is influenced by multiple extrinsic and intrinsic factors (85). Extrinsic factors include smoking, alcohol, and certain use of medicines While intrinsic factors cover injury to the periosteum or endosteum and poor vascularization at the injury site (86). Although we have achieved great improvement in surgical techniques nowadays, sometimes osteoporotic fractures-related impaired bone regeneration like non-union would happen, which greatly affects the life quality of a number of patients (87). Therefore, it is of great need to do intensive investigations to fill the clinical gaps and develop effective therapeutic approaches for these affected patients.

In general, fracture healing is subdivided into four biological phases followed in a chronological order: hematoma formation and inflammatory response, proliferation and differentiation, ossification, and remodeling (88). These phases are involved in many physiological processes, like inflammation, angiogenesis, stem cell differentiation, osteogenesis, and chondrogenesis with the involvement of various types of bone-related cells (89). Exosomes are closely connected with these processes through mediating cell-to-cell communications (**Table 3**). In fact, exosomes could promote osteoblasts’ differentiation, suppress osteoclasts formation and differentiation, and accelerate angiogenesis. In addition, some important intrinsic factors mentioned above could modify the functions of exosomes to regulate the healing process. BM-MSCs could be induced to recruit, proliferate, migrate, and differentiate into osteoblasts and chondrocytes during intramembranous ossification and endochondral ossification, which played important roles in osteoporotic fracture healing (99). Reliable evidence has illustrated the capacities of osteogenic differentiation for BM-MSCs-derived exosomes. For example, Furuta et al. used the CD9^–/–^ mouse, an established model with reduced levels of exosomes, and found there was an obvious delay of endochondral ossification and fracture healing compared with the wild-type mouse. More interestingly, the delay could be rescued by injecting BM-MSCs-exosomes (90). Besides, Narayanan et al. incubated exosomes isolated from osteogenic differentiated BM-MSCs with BM-MSCs, finding BM-MSCs internalized these exosomes and gave rise to an extensive upregulation of several important genes like bone morphogenetic protein 9 (BMP9), transforming growth factor β1 (TGFβ1), transcription factors, and ECM molecules (50). Xu et al. showed similar results and detected this impact was involved in the modulatory effect of miRNAs on target genes and pathways (100). Among these, miR-196a acted as one of the most significant molecules in the modulatory process and miR-21 and miR-25 have also proven to possess the ability to accelerate osteogenesis and angiogenesis (90, 91). More importantly, BM-MSCs-exosomes contained a variety of bone repair-related cytokines such as monocyte chemotactic protein 1 (MCP-1), monocyte chemotactic protein 3 (MCP-3), stromal cell-derived factor-1 (SDF-1), and angiogenic factors, which could promote fracture healing (101). Recently, some researchers transplanted BM-MSCs-derived exosomes into the fracture site in a rat model of femoral non-union, obtaining the results that these exosomes could significantly trigger osteogenesis and angiogenesis to promote bone healing process though activating the BMP-2/Smad1/RUNX2 and the HIF-1α/VEGF signaling pathways (92). Furthermore, some studies also revealed that exosomes secreted by human umbilical cord mesenchymal stem cells (uMSCs) could enhance angiogenesis to accelerate bone healing by overexpressing VEGF, HIF‐1α (93), or Wnt signaling pathway (94), which provided a new view for us regarding uMSCs-related bone fracture. As we mentioned above, some intrinsic factors could change the functions of exosomes and thus affect the osteoporotic fracture healing. Xu et al. observed that aged exosomes had obvious attenuated effects on MSCs osteogenic differentiation *in vitro* and facture healing *in vivo*. Under further investigation, they found miR-128-3p was dramatically over-expressed in aged-BM-MSCs-derived exosomes, which functioned as a suppressor in the process of fracture healing by directly targeting the 3’-UTR of Smad5 (95). Hence, exosomal miR-128-3p antagomir could be an ideal treatment for bone fracture healing, especially for the elderly. Wang et al. observed that the healing time was longer in obese fracture patients than normal weight patients. Interestingly, using bioinformatics analysis and related assays, they further found that high-fat treatment could decrease the secretion of BM-MSCs-derived exosomes and reduce the carried lncRNA H19 *via* miR-467/HoxA10 axis, hence affecting osteogenic differentiation and fracture (96). Liu et al. performed a serious of *in vivo* and *in vitro* experiments to verify that hypoxia preconditioning BM-MSCs-derived exosomes exerted a greater effect on promoting fracture healing. Hypoxic MSC-derived exosomes were enriched with miR-126, which possessed the abilities of being pro-angiogenesis, pro-proliferative, and pro-migratory by suppressing SPRED1/Ras/Erk signaling pathway. Therefore, hypoxia-induced BM-MSCs transplantation might have great potential as a therapy for fracture, but several challenges remain to be overcome to achieve clinical applications (97). Moreover, a study by Xiong et al. explored the function of M2 macrophages in bone formation and its underlying mechanisms. They co-cultured M2 macrophages and BM-MSCs and isolated the exosomes secreted by M2 macrophages, finding M2 macrophages-derived exosomes (M2D-Exos) were rich in miR-5106, which could be internalized by BM-MSCs and promote osteoblasts’ differentiation to accelerate healing *via* targeting the osteogenic related genes, like Salt-inducible kinase 2 and 3 (SIK2 and SIK3). Therefore, local injection of M2D-Exos might be a significant therapeutic strategy to accelerate bone fracture healing (98).

**Table 3:** Exosomes in fracture healing.

Origin of exosomes	Exosomes contains	Recipient cell	Involved pathway	Function	Reference
MSCs	MiR-196a, miR-21	Not referred	Not referred	Promoted bone healing	(90)
BM-MSCs	MiR-25	Osteoblasts	(SMURF1), Runx2	Accelerated osteogenic differentiation, proliferation, and migration of osteoblasts	(91)
BM-MSCs	Not referred	HUVECs and MC3T3-E1	BMP-2/Smad1/RUNX2 and the HIF-1α/VEGF signaling pathways	Enhanced osteogenesis, angiogenesis	(92)
uMSCs	Not referred	HUVECs	HIF-1α	Promoted angiogenesis	(93)
uMSCs	Not referred	Not referred	Wnt signaling pathway	Accelerated bone healing	(94)
Aged-BM-MSCs	MiR-128-3p	MSCs	Smad5	Inhibited bone healing	(95)
High-fat treatment BM-MSCs	LncRNA H19	Osteoblasts	MiR-467/HoxA10 axis	Inhibited osteogenesis	(96)
Hypoxia preconditioning BM-MSCs	MiR-126 and	MSCs	the SPRED1/Ras/Erk signaling pathway	Promoted bone healing	(97)
M2 macrophage	MiR-5106	Osteoblasts	SIK2 and SIK3	Promoted osteoblast differentiation	(98)

SMURF1, Smad ubiquitination regulatory factor 1; SIK2, Salt-inducible kinase 2; SIK3, Salt-inducible kinase 3.

Current studies provided powerful evidence that exosomes appeared to show the ability to enhance osteogenesis and angiogenesis to promote fracture healing *via* multiple pathways, implying a novel insight to comprehend the process of bone regeneration. Therefore, we need to spare no effort to do related research and detect an effective treatment for osteoporotic fracture.

## Summary and Perspectives

**Figure 2:**
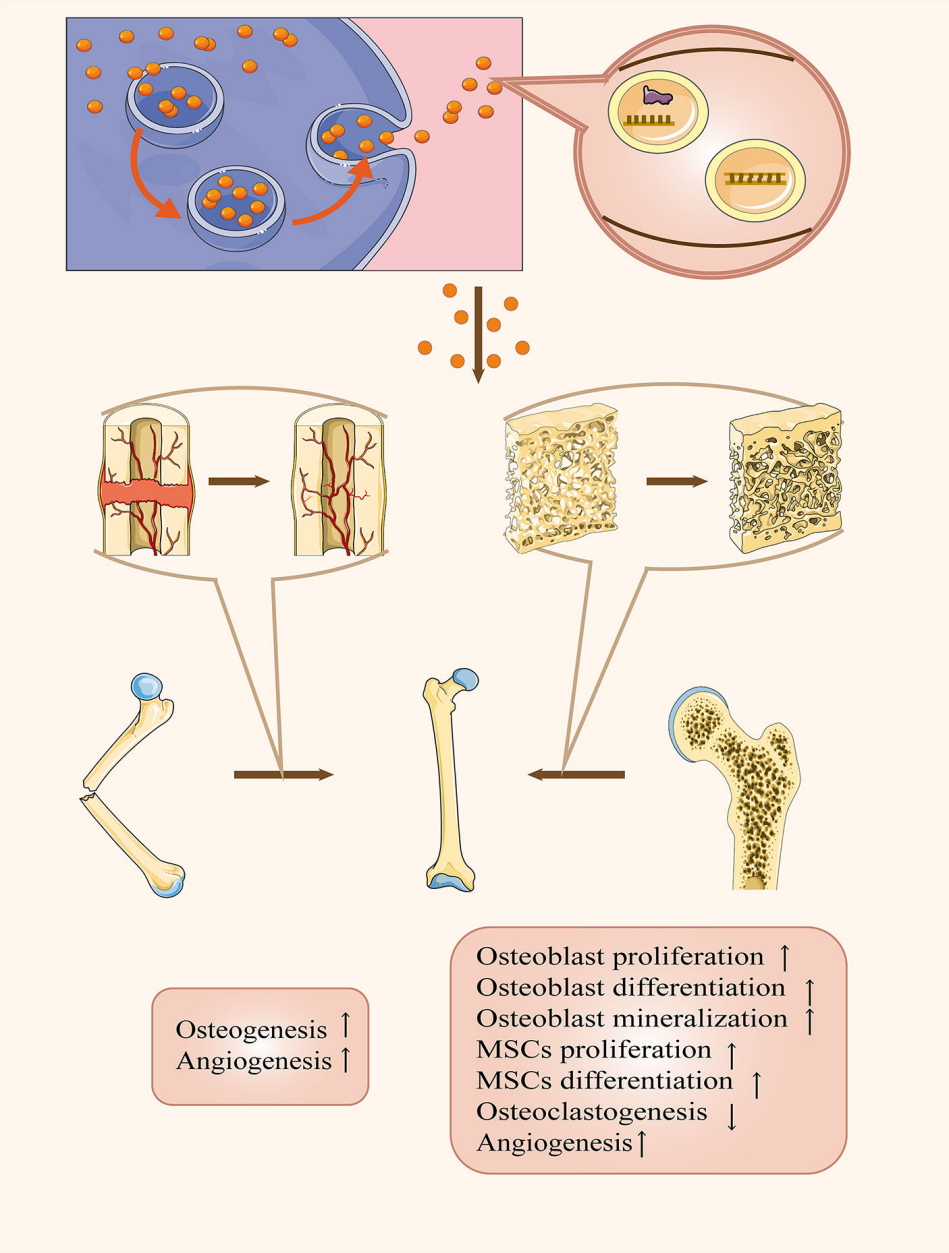
The therapeutic applications of exosomes in OP and related fracture healing. Exosomes can act as therapeutic agents or drug carriers not only to remit OP by facilitating osteoblast proliferation, differentiation, mineralization, and MSCs proliferation, differentiation and angiogenesis as well as suppressing osteoclastogenesis but also accelerating fracture healing via promoting osteogenesis and angiogenesis.

The number of people with OP is increasing rapidly among generations, coupled with a growing number of osteoporotic fractures (102). Moreover, osteoporotic fractures increase the incidence of abnormal fracture healing, like delayed healing or non-union, which aggravates the heavy burden placed on healthcare systems as well as the economy. Hence, we should endeavor to look for more effective ways to prevent and treat osteoporotic fractures. Nowadays, the application of exosomes in osteoporotic fractures has received much attention, and lots of scientists have revealed multiple functions of exosomes in this field. As outlined above, exosomes played an essential role in mediating osteoporotic fractures. First, many researchers found obvious dysregulations of certain contents in circulatory exosomes from patients with OP or related impaired fracture healing, which could be promising biomarkers for the diagnosis of OP. Second, exosomes were a double-edged sword for OP by transporting their cargos to modify the activities of surrounding or distant bone-related cells. Third, exosomes have been applicated as a brilliant drug delivery system to treat osteoporotic fractures by accelerating osteogenesis and angiogenesis (**Figure 2**). However, the specific mechanisms of intercellular communications *via* exosomes is still not fully understood. A recent study by Ge et al. detected 1536 proteins contained in osteoblasts-derived exosomes and 172 among them overlapped with proteins in the bone database, such as ephrinB1 (EFNB1), transforming growth factor beta receptor 3 (TGFBR3), lipoprotein receptor-related protein (LRP6), bone morphogenetic protein receptor type-1 (BMPR1), and Smad ubiquitylation regulatory factor-1 (SMURF1) (103). Besides, increasing data unveiled there were nine overexpressed miRNAs (let-7a, miR-199b, miR-218, miR-148a, miR-135b, miR-203, miR-219, miR-299-5p, and miR-302b) and four down-regulated (miR-221, miR-155, miR-885-5p, miR-181a, and miR-320c) in MSCs-derived exosomes (100). However, these complicated mechanisms and the therapeutic potential for osteoporotic fractures remain a mystery and there is a clear need for us to perform further investigations. On the other hand, Xie et al. found that BM-MSCs-derived exosomes could promote bone formation when mixed with decalcified bone matrix scaffolds (104). But it is still a serious challenge to quantify and separate different exosome subpopulations as well as modify these exosomes for us. Overall, it should be noted that several significant developments are expected to occur, including: (*a*) clarifying the specific mechanisms of cargos within bone-related cells-derived exosomes in OP and osteoporotic fracture; (*b*) elucidating the functions of exosomes in the occurrence and healing of osteoporotic fractures, which possess both positive and negative effects; (*c*) discovering credible biomarkers contained in circulatory exosomes of clinical significance in the early diagnosis of OP; (*d*) improving the methodologies of extraction and separation of exosomes; and (*e*) propelling clinical application in the treatment of osteoporotic fracture through utilizing them as therapeutic agents or drug carriers. Advances in these areas will likely require new experimental techniques, superior creativity, and much work. But at the same time, such advances will help us to get a more comprehensive understanding of osteoporotic fractures and allow scientists to translate this knowledge into exosome-based therapies and diagnosis in clinic. Therefore, exosomes provide a promising method to improve the therapeutic effects of osteoporotic fractures, even though an increase in research is demanded.

## Author Contributions

ZY: Writing - Original Draft and Editing. WZ: Writing - Review and Editing, Visualization. XR: Writing - Original Draft. CT: Writing - Review and Editing, Supervision, Funding acquisition. ZL: Writing - Review and Editing, Supervision, Funding acquisition. All authors contributed to the article and approved the submitted version.

## Funding

This work was supported by the National Natural Science Foundation of China (No. 81902745), Hunan Provincial Research and Development Program in Key Areas (2019WK2071, 2020DK2003), and China Postdoctoral Science Foundation (No. 2021M693557).

## Conflict of Interest

The authors declare that the research was conducted in the absence of any commercial or financial relationships that could be construed as a potential conflict of interest.

The handling editor declared a shared affiliation with the authors at time of review.
